# Dietary quercetin and glucoraphanin supplementation improves growth performance and modulates ruminal microbiota in meat sheep

**DOI:** 10.3389/fvets.2026.1976936

**Published:** 2026-10-08

**Authors:** Rao Li, Xiuyuan Yu, Huimei Feng, Chaowu Fu, Yurong Yang, Yibao Jiang

**Affiliations:** 1 College of Animal Science and Technology, Henan Agricultural University, Zhengzhou, China; 2 College of Veterinary Medicine, Henan Agricultural University, Zhengzhou, China

**Keywords:** glucoraphanin, growth performance, meat sheep, quercetin, ruminal microbiota

## Abstract

**Introduction:**

This study investigated the effects of single or combined dietary supplementation with quercetin (QUE) and glucoraphanin (GRA) on growth performance, serum antioxidant capacity and biochemical parameters, rumen fermentation, and ruminal microbiota of meat sheep.

**Methods:**

A total of 60 meat sheep (3–4 months old) with similar body weights were randomly divided into four groups, with three replicates per group and five sheep per replicate, and the feeding trial lasted for 35 days. Sheep in the control group (CON) were fed a basal diet. The TG1 group was supplemented with 300 mg/kg QUE; the TG2 group with 200 mg/kg GRA, and the TG3 group with 300 mg/kg QUE plus 200 mg/kg GRA. All additives were supplemented externally without altering the nutritional composition of the basal diet.

**Results and discussion:**

The results showed that dietary supplementation with quercetin (QUE) and glucoraphanin (GRA) exerted a significant interactive effect on average daily feed intake (ADFI) of meat sheep (*p* < 0.05). Serum biochemical analysis revealed a significant interaction between QUE and GRA for serum ALT levels (*p* < 0.05). In addition, analysis of serum antioxidant indices indicated that QUE and GRA had a significant interactive effect on serum GSH-Px activity (*p* < 0.05). Notably, significant interactive effects of QUE and GRA were also observed on the relative abundances of *Succiniclasticum* and *Ruminococcus* among ruminal microorganisms (*p* < 0.05). In summary, dietary supplementation of QUE and GRA improved the antioxidant capacity of meat sheep. Changes in serum indicators suggested that they might exert potentially beneficial effects on liver function. Meanwhile, the co-supplementation could modulate the relative abundance of several ruminal bacterial genera and produce certain beneficial effects on partial growth-performance and meat quality indicators of meat sheep, which provides preliminary evidence for the combined application of QUE and GRA in meat sheep production.

## 1 Introduction

High-concentrate diets can boost economic value in meat sheep production, but they can cause rumen dysbiosis, aggravated oxidative stress, and impaired immune function (1). Concurrently, the misuse of antibiotics in rapidly expanding livestock sectors has led to bacterial resistance and greater methane emissions, threatening global public health and agricultural sustainability (2, 3). There is thus an urgent need to identify and apply safe, effective natural extracts as antibiotic alternatives.

Quercetin (QUE) is a flavonol compound widely present in plants such as onions, apples, and green tea. As a natural bioactive compound, quercetin exhibits multiple beneficial effects, including antioxidant, anti-inflammatory, and gut microbiota-modulating properties (4). Studies have shown that dietary QUE supplementation in broilers improves growth performance, intestinal morphology, and the composition of the gut microbiota (5). In addition, QUE influences rumen fermentation parameters and ruminal bacterial communities *in vitro*, reducing methane emissions (6). Previous reports have revealed that QUE modulates signaling pathways including Nrf2 and NF-κB, enhances cellular antioxidant defense, and mitigates inflammatory reactions (7). Glucoraphanin (GRA) is an important glucosinolate present in cruciferous vegetables, with broccoli particularly rich in glucosinolates; GRA is the predominant component, accounting for approximately 50% of the total glucosinolate content (8). GRA itself has no direct biological activity; it is hydrolyzed by the intestinal or ruminal microbiota and converted into sulforaphane (SFN), which has stronger bioactivity. SFN exhibits antibacterial, anti-inflammatory, and anti-cancer properties and plays a vital role in maintaining host health (9). As a hydrolytic metabolite of GRA, SFN exerts antioxidant, anti-inflammatory and anti-apoptotic activities via regulating the endoplasmic reticulum stress pathway, and thus protects against cardiovascular diseases, neurological disorders, diabetes and cancers (10). Previous reports have demonstrated that sulforaphane (SFN) can activate the Nrf2 signaling pathway, exert prominent antioxidant and anti-inflammatory effects, and effectively alleviate chronic oxidative stress and inflammatory damage in the body. It also possesses the capacity to enhance immune function and modulate gut microbiota (11, 12). However, most existing studies have only explored the effects of quercetin or glucoraphanin individually, and most experiments were carried out using monogastric animal models such as mice. In-depth research on their combined effects and application potential in ruminants is still lacking. Given their functional complementarity: QUE can directly scavenge reactive oxygen radicals in the organism, and its phenolic metabolites can selectively modulate the structural composition of the ruminal microbial community. In contrast, SFN, the metabolite produced by microbial hydrolysis of GRA, promotes endogenous antioxidant and anti-inflammatory responses in host cells via pathways including Nrf2. Accordingly, we hypothesize that dietary supplementation with QUE and GRA may exert an interactive effect and achieve more comprehensive protective outcomes compared with single supplementation.

To test the above hypothesis, this study was designed to investigate the effects of dietary supplementation with QUE and GRA on growth performance and rumen health in meat sheep, and to examine the interaction between the two compounds, thereby providing a theoretical basis for the green and sustainable development of meat sheep production.

## 2 Materials and methods

### 2.1 Animal breeding and experimental design

Quercetin (QUE, purity ≥98%, raw powder containing no extra carriers, excipients or other auxiliary materials) and glucoraphanin (GRA, purity ≥95%, raw powder containing no extra carriers, excipients or other auxiliary materials) were purchased from Liyuan Biotechnology Center (Zhengzhou, Henan, P.R. China). Dietary inclusion levels in this study represent the target contents of active ingredients. This feeding trial was conducted at Longxupo Agriculture and Animal Husbandry Co., Ltd., Luoyang City, Henan Province, P.R. China, from August 5 to September 15, 2025. During the experimental period, the temperature inside the sheep barn ranged from 25 to 30 °C with an average relative humidity of 55%−65%. The barn was ventilated via a combination of natural ventilation and mechanical exhaust fans. All sheep received unified deworming and immunization procedures prior to the trial. Twenty-one days before the adaptation period, each sheep received a 3 mL subcutaneous injection in the neck of inactivated *Mycoplasma ovipneumoniae* vaccine (Hefei Qiangli Animal Pharmaceutical Co., Ltd., Hefei, China). Seven days before the start of the adaptation period, sheep were orally administered ivermectin-oxibendazole powder (Chongqing Aolong Biological Products Co., Ltd., Chongqing, China) at 100 mg/kg body weight (calculated as the preparation) to eliminate internal and external parasites. Animals exhibiting lameness, cough, persistent diarrhea, or poor coat condition were excluded from the experiment. Finally, 60 healthy Dorper × Hu crossbred rams aged 3–4 months with similar initial body weights (39.49 ± 0.85 kg) were randomly allocated into four groups, with three replicates per group and five sheep per replicate. Sheep in the control group (CON) were fed a basal diet, while the TG1 group was supplemented with 300 mg/kg QUE, the TG2 group with 200 mg/kg GRA, and the TG3 group with a combination of 300 mg/kg QUE and 200 mg/kg GRA. The basal diet was formulated to meet the nutrient requirements for sheep according to NRC (2007). For BW and ADG, each sheep was an independent experimental unit (15 biological replicates per group). For other indices, one sheep selected from each replicate served as an independent experimental unit for slaughter and sampling, with a total of three biological replicates per group. The dosage of QUE was determined with reference to the effective doses reported in sheep studies, as well as doses proven to improve production performance, optimize intestinal microbiota and remodel small intestinal morphology in poultry trials (13, 14). The supplemental dosage of GRA was established based on verified effective dosages and maximum safe dosage levels documented in mouse and rat studies (15, 16). All sheep were fed twice daily at 05:30 and 17:30, following the farm's standard management protocol.

In this experiment, quercetin (QUE) and glucoraphanin (GRA) powders were first premixed with the premix carrier by geometric dilution to ensure homogeneous distribution of trace components. Corn was ground through a 2 mm sieve in an integrated feed crushing-mixing machine (Xingyang Minsheng Machinery Factory, China), and then thoroughly mixed with soybean meal and the QUE/GRA premix to produce the concentrate. Peanut vine hay was fed separately, and the concentrate and peanut vine hay were offered in different feed troughs. The composition and nutrient levels of the basal diet for the sheep are presented in Table 1.

**Table 1:** Basal diet composition and nutritional levels (on a dry matter basis).

Ingredients	Content (%)	Nutrient levels	Content
Corn	49.80	Digestible energy (MJ/kg)	10.11
Soybean meal	16.80	Crude protein (%)	14.21
Peanut vine hay	30.00	Ash (%)	5.21
NaCl	0.60	Ether extract (%)	6.07
Premix^a^	2.80	Crude fiber (%)	13.66
Total	100	Neutral detergent fiber (%)	41.80
		Acid detergent fiber (%)	24.47
		Calcium (%)	1.02
		Phosphorus (%)	0.44

Premix^a^ supplied per kilogram of the basal diet: vitamin A 8,200 IU; vitamin D_3_ 3,700 IU; vitamin E 20 mg; Fe 90 mg; Zn 100 mg; Cu 15 mg; I 1.5 mg; Mn 52 mg; Se 0.35 mg; Co 0.16 mg. Crude protein (DM basis): concentrate, 19.60%; peanut vine hay, 9.55%. The overall dietary nutrient values in this table were calculated by weighting the measured values of concentrate and peanut vine hay according to their feeding proportions.

### 2.2 Determination of growth performance

The body weights of the sheep were recorded at the end of the 7-day adaptation period (the initiation of the formal trial). At the end of the 35-day feeding period, all sheep were weighed following a 12-h fast to determine the final body weight. Daily feed allocation for each group was recorded throughout the trial. Residual feed from the previous day was collected and weighed prior to morning feeding. The data were used to calculate the average daily feed intake (ADFI), average daily gain (ADG), and feed-to-gain ratio (F/G).

### 2.3 Determination of muscle quality and nutrient composition

At the end of the experiment, one sheep was randomly selected for slaughter from each of the three replicate pens per group (three sheep per group in total). Animals were exsanguinated and slaughtered, and approximately 1 kg of longissimus dorsi muscle was collected. Each sample was measured in triplicate. Physicochemical analysis of muscle was performed according to standard methods, including muscle pH, drip loss, cooking loss and muscle nutrient composition (17, 18).

### 2.4 Determination of serum biochemical indicators

At the end of the trial, one sheep was randomly selected for blood collection from each of the three replicate pens per group (three sheep per group in total). After a 12 h fasting period, approximately 3–5 mL of blood was drawn from the jugular vein, placed into procoagulant tubes, and allowed to clot before centrifugation at 3,500 rpm for 10 min at 4 °C. After centrifugation, the supernatant was collected, transferred to 1.5-mL centrifuge tubes, and stored at −80 °C until analysis. Serum biochemical parameters were measured using a BK280 fully automatic biochemical analyzer (BIOBASE Co., Ltd., Jinan, China) with commercial assay kits according to the manufacturer's instructions. The measured parameters were alanine aminotransferase (ALT), aspartate aminotransferase (AST), albumin (ALB), blood urea nitrogen (BUN), triglyceride (TG), total cholesterol (TC), lactate dehydrogenase (LDH), total protein (TP), and globulin (GLB).

### 2.5 Determination of serum antioxidant indicators

Blood samples were processed and preserved according to the method described in Section 2.4. Serum antioxidant indices, including total antioxidant capacity (T-AOC), total superoxide dismutase (T-SOD), malondialdehyde (MDA), and glutathione peroxidase (GSH-Px), were determined using commercial assay kits (Nanjing Jincheng Bioengineering Institute, Nanjing, China) following the manufacturer's protocols. The brief procedures were as follows: T-AOC was measured using the ABTS method at 405 nm (catalog no. A015-2-1); T-SOD activity was determined by the WST-1 method at 450 nm (catalog no. A001-3); MDA content was detected using the thiobarbituric acid (TBA) method at 532 nm (catalog no. A003-1); GSH-Px activity was assayed with the DTNB colorimetric method at 412 nm (catalog no. A005-1).

### 2.6 Rumen fermentation parameters

After slaughter (one sheep slaughtered from each of the three replicate pens per group, three sheep per group in total), the rumen contents were collected, and the rumen fluid was filtered through four layers of sterile gauze. The pH of the rumen fluid was measured immediately after collection using a portable pH meter (PHBJ-260, Leici, Shanghai, China), with three replicate measurements per sample, and the mean value was calculated. The remaining rumen fluid was temporarily stored in liquid nitrogen and transferred to a −80 °C freezer for further analysis. Ruminal ammonia nitrogen (NH_3_-N) was measured by the modified phenol-hypochlorite colorimetric method (19). Volatile fatty acids (VFA) were determined using an ion chromatograph (Thermo Scientific Dionex ICS-5,000+, USA) equipped with an IonPac AS11-HC analytical column (250 mm × 4 mm) and an AERS-4 mm suppressed conductivity detector. The detector temperature was 35.00 °C, the initial column oven temperature was 30.00 °C, and the autosampler temperature was 25.00 °C. The mobile phase flow rate was 1.000 mL/min, the injection volume was 25 μL, and the suppressor operating current was 13 mA. A gradient elution program was applied, and the total instrument run time for a single injection was 22.10 min. A mixed VFA external standard (Sigma-Aldrich, USA) was used to prepare serial gradient concentrations for analyte identification and quantification via the external standard method. For sample preparation, the filtered rumen fluid was centrifuged at 12, 000 rpm for 5 min at 4 °C. Then, 500 μL of the supernatant was mixed with 100 μL of 25% metaphosphoric acid solution and kept at 4 °C for 24 h. After a second centrifugation, the supernatant was manually diluted 100-fold with ultrapure water, filtered through a 0.22 μm aqueous membrane filter, and then subjected to instrumental analysis.

### 2.7 Analysis of the rumen microbiota

One sheep was randomly selected from each replicate for slaughter, and rumen contents were collected for 16S rRNA sequencing, yielding a total of three sequencing samples per group. Total microbial DNA was extracted from samples using SDS-based lysis buffer combined with proteinase K digestion. The hypervariable V3-V4 region of the 16S rRNA gene was amplified with Phusion high-fidelity DNA polymerase using the primers 338F (5′-ACTCCTACGGGAGGCAGCAG-3′) and 806R (5′-GGACTACHVGGGTWTCTAAT-3′). Sequencing libraries were constructed with the KAPA library preparation kit and sequenced on the Illumina NovaSeq platform. Library quality control and concentration normalization were performed using the Qubit 4.0 fluorometer, and each sample generated no fewer than 50 000 valid sequencing reads. FastQC was used for quality evaluation of raw sequencing data. Trimmomatic was applied to filter low-quality sequences (*Q* < 20), and Cutadapt was used to remove primer sequences. Subsequently, the DADA2 algorithm was used for sequence denoising and chimera removal to obtain amplicon sequence variants (ASVs). Taxonomic annotation of ASVs was performed using the RDP Classifier against the SILVA database (version 138.1). Rarefaction curves were plotted to evaluate whether the sequencing depth was sufficient to cover microbial species in the samples. Alpha-diversity indices including the Chao1, Shannon and Simpson indices were calculated based on the ASV abundance table to reflect species richness and community diversity of the microbial community. Two-way analysis of variance (ANOVA) was used to analyze the main and interactive effects of treatments on genus-level bacterial relative abundance. The Benjamini–Hochberg method was adopted for FDR multiple-testing correction, and adjusted *p* < 0.05 was regarded as statistically significant. Beta-diversity analysis was performed based on the Bray–Curtis distance matrix. Principal coordinate analysis (PCoA) was used for community-structure visualization, and an ANOSIM test was conducted to assess differences in community structure among groups.

### 2.8 Statistical analysis

Experimental raw data were sorted and preliminarily calculated using Microsoft Excel 2019. Statistical analyses were performed with SPSS 26.0.(IBM Corporation, Armonk, NY, USA). The Shapiro–Wilk test and Levene's test were used to assess the normality and homogeneity of variance of model residuals, respectively. ADFI and F/G were analyzed by two-way ANOVA with pen as the experimental unit (*n* = 3 per treatment) to evaluate the main effects of QUE, GRA and their interaction. For BW and ADG, individual sheep served as the observational unit (*n* = 15 per treatment). Given that animals were nested within pens, a linear mixed-effects model was adopted, in which QUE, GRA and their interaction were set as fixed effects and pen as a random effect to correct for clustering correlation among sheep within the same pen. Slaughter and sampling-related indicators were analyzed via two-way ANOVA, with one sheep selected from each pen (*n* = 3 per treatment). All results are expressed as means ± standard deviation (SD). Statistical significance was defined as *p* < 0.05.

## 3 Results

### 3.1 Growth performance

The effects of different treatments on growth performance are presented in Table 2. The main effect of QUE was significant for increasing ADG and decreasing the F/G ratio (*p* < 0.05). The main effect of GRA significantly reduced ADFI and the F/G ratio (*p* < 0.05). In addition, there was a significant interaction between QUE and GRA for ADFI (*p* < 0.05).

**Table 2:** Effects of dietary supplementation with quercetin and glucoraphanin on the growth performance of meat sheep.

Item	Groups				*p* value		
	CON	TG1	TG2	TG3	TG1	TG2	TG1 × TG2
IBW, kg	39.16 ± 0.63	39.32 ± 0.81	39.74 ± 1.10	39.38 ± 0.79	0.650	0.154	0.247
FBW, kg	46.42 ± 1.26	46.93 ± 1.58	47.02 ± 1.60	47.56 ± 1.60	0.184	0.121	0.980
ADG, g/d	172.70 ± 23.88	181.27 ± 31.66	173.49 ± 25.91	194.76 ± 28.63	0.041	0.322	0.378
ADFI, g/d	1,973.80 ± 90.42	1,874.22 ± 112.58	1,827.48 ± 70.5	1,876.01 ± 106.22	0.346	0.039	0.031
F/G	11.48 ± 0.53	10.35 ± 0.62	10.56 ± 0.41	9.62 ± 0.54	< 0.001	< 0.001	0.321

For ADFI and F/G, *P* values were obtained from two-way ANOVA to evaluate the main effects of QUE (TG1), GRA (TG2), and their interaction (TG1 × TG2). For BW and ADG, *P* values were derived from a linear mixed-effects model with pen as a random effect to test the same main and interactive effects. Differences were considered statistically significant at *p* < 0.05. IBW, initial body weight; FBW, final body weight; F/G, feed-to-gain ratio. ADFI and F/G used pen as the experimental unit (*n* = 3). BW and ADG used individual sheep as the experimental unit (*n* = 15).

### 3.2 Muscle quality and nutrient composition

Muscle quality and nutrient composition data are presented in Table 3. The main effects of QUE and GRA were significant for increasing muscle pH at 24 h (*p* < 0.05). In addition, the main effect of QUE significantly decreased the ether extract content in muscle (*p* < 0.05).

**Table 3:** Effects of dietary supplementation with quercetin and glucoraphanin on the muscle quality and nutrient composition of meat sheep (*n* = 3 per group).

Item	Groups				*p* value		
	CON	TG1	TG2	TG3	TG1	TG2	TG1 × TG2
pH_45min_	6.21 ± 0.27	6.30 ± 0.03	6.27 ± 0.17	6.43 ± 0.23	0.308	0.434	0.776
pH_24h_	5.67 ± 0.16	5.99 ± 0.03	6.06 ± 0.19	6.23 ± 0.18	0.026	0.007	0.417
Drip loss (%)	7.11 ± 0.77	6.65 ± 3.85	7.08 ± 0.72	6.45 ± 0.75	0.654	0.926	0.946
Cooking loss (%)	24.75 ± 0.72	24.54 ± 0.79	23.88 ± 0.91	23.97 ± 0.49	0.892	0.140	0.745
Crude protein (%)	81.71 ± 1.47	82.57 ± 2.06	81.80 ± 1.00	82.21 ± 1.96	0.533	0.894	0.824
Ether extract (%)	14.55 ± 2.78	12.10 ± 2.67	13.84 ± 2.41	9.43 ± 1.16	0.035	0.248	0.490

*p* values are obtained from two-way ANOVA for the main effects of QUE (TG1), GRA (TG2), and their interaction (TG1 × TG2). Differences were considered statistically significant at *p* < 0.05. Crude protein and ether extract of the *longissimus dorsi* muscle are presented on a dry-matter basis (% DM).

### 3.3 Serum biochemical indices

Serum biochemical indices are presented in Table 4. The main effect of QUE was significant for reducing LDH concentration (*p* < 0.05). In addition, there was a significant interaction between QUE and GRA for ALT concentration (*p* < 0.05).

**Table 4:** Effects of dietary supplementation with quercetin and glucoraphanin on serum biochemical indices of meat sheep (*n* = 3 per group).

Item	Groups				*p* value		
	CON	TG1	TG2	TG3	TG1	TG2	TG1 × TG2
BUN (mmol/L)	5.20 ± 0.58	4.50 ± 0.15	5.81 ± 1.75	5.14 ± 0.82	0.273	0.316	0.974
TP (g/L)	75.50 ± 9.10	80.00 ± 5.73	81.60 ± 6.24	77.90 ± 9.43	0.931	0.669	0.389
GLB (g/L)	34.13 ± 2.31	37.83 ± 1.17	36.40 ± 1.66	37.43 ± 2.82	0.085	0.416	0.301
ALB (g/L)	34.27 ± 2.55	31.53 ± 2.37	32.53 ± 2.93	32.43 ± 1.81	0.346	0.776	0.379
TG (mmol/L)	0.26 ± 0.04	0.21 ± 0.02	0.20 ± 0.04	0.18 ± 0.04	0.134	0.062	0.631
TC (mmol/L)	1.40 ± 0.04	1.34 ± 0.03	1.35 ± 0.21	1.29 ± 0.08	0.418	0.473	1.000
LDH (U/L)	765.90 ± 68.84	694.23 ± 24.88	722.03 ± 54.26	664.63 ± 8.27	0.047	0.219	0.802
ALT (U/L)	17.07 ± 2.21	9.77 ± 2.18	10.83 ± 3.35	11.53 ± 2.20	0.054	0.166	0.026
AST (U/L)	180.57 ± 30.99	173.50 ± 24.58	173.00 ± 28.01	170.57 ± 11.51	0.750	0.725	0.876

*p* values are obtained from two-way ANOVA for the main effects of QUE (TG1), GRA (TG2), and their interaction (TG1 × TG2). Differences were considered statistically significant at *p* < 0.05. BUN, blood urea nitrogen; TP, total protein; GLB, globulin; ALB, albumin; TG, triglyceride; TC, total cholesterol; LDH, lactic dehydrogenase; ALT, alanine transaminase; AST, aspartate aminotransferase.

### 3.4 Serum antioxidant capacity

Serum antioxidant capacity data are presented in Table 5. The main effect of QUE was significant for increasing serum SOD and GSH-Px levels (*p* < 0.05). In addition, there was a significant interaction between QUE and GRA for serum GSH-Px levels (*p* < 0.05).

**Table 5:** Effects of dietary supplementation with quercetin and glucoraphanin on serum antioxidant capacity of meat sheep (*n* = 3 per group).

Item	Groups				*p* value		
	CON	TG1	TG2	TG3	TG1	TG2	TG1 × TG2
T-AOC (U/mL)	0.93 ± 0.10	0.90 ± 0.04	0.92 ± 0.09	1.00 ± 0.08	0.526	0.401	0.269
SOD (U/mL)	62.12 ± 2.78	66.30 ± 1.70	65.04 ± 1.62	66.29 ± 1.36	0.042	0.231	0.229
MDA (nmol/mL)	4.81 ± 0.80	4.07 ± 0.58	4.53 ± 0.48	3.96 ± 0.60	0.111	0.609	0.815
GSH-Px (U/mL)	253.79 ± 4.77	271.72 ± 22.79	251.03 ± 10.41	306.21 ± 4.13	0.001	0.066	0.037

*p* values are obtained from two-way ANOVA for the main effects of QUE (TG1), GRA (TG2), and their interaction (TG1 × TG2). Differences were considered statistically significant at *p* < 0.05. T-AOC, total antioxidant capacity; SOD, superoxide dismutase; MDA, malondialdehyde; GSH-Px, glutathione peroxidase.

### 3.5 Rumen fermentation parameters

Rumen fermentation parameters data are presented in Table 6. The main effect of QUE was significant for decreasing ruminal NH_3_-N concentration and increasing the concentrations of ruminal acetate and total volatile fatty acids (TVFA; *p* < 0.05). The main effect of GRA was significant for the concentrations of ruminal acetate, propionate and TVFA (*p* < 0.05).

**Table 6:** Effects of dietary supplementation with quercetin and glucoraphanin on rumen fermentation parameters of meat sheep (*n* = 3 per group).

Item	Groups				*p* value		
	CON	TG1	TG2	TG3	TG1	TG2	TG1 × TG2
NH_3_-N (mg/dL)	20.88 ± 1.78	16.79 ± 1.24	18.54 ± 1.10	15.88 ± 1.19	0.003	0.072	0.386
pH	7.20 ± 0.24	6.99 ± 0.08	6.59 ± 0.69	6.95 ± 0.09	0.722	0.169	0.212
Acetic acid (mmol/L)	18.37 ± 2.47	24.85 ± 0.47	24.63 ± 1.40	27.45 ± 1.37	0.001	0.001	0.082
Propionic acid (mmol/L)	7.40 ± 1.53	9.86 ± 1.30	11.91 ± 1.66	12.99 ± 1.5	0.104	0.002	0.215
Butyric acid (mmol/L)	3.51 ± 1.36	3.60 ± 1.06	4.75 ± 1.10	5.33 ± 0.94	0.626	0.052	0.716
Isobutyric acid (mmol/L)	0.60 ± 0.06	0.57 ± 0.06	0.58 ± 0.03	0.63 ± 0.07	0.739	0.473	0.268
Isovaleric acid (mmol/L)	0.48 ± 0.02	0.50 ± 0.03	0.51 ± 0.02	0.52 ± 0.01	0.444	0.054	0.468
Valeric acid (mmol/L)	0.77 ± 0.07	0.83 ± 0.11	0.81 ± 0.05	0.87 ± 0.04	0.210	0.397	0.954
Total volatile fatty acids (mmol/L)	41.11 ± 1.62	51.29 ± 3.76	56.46 ± 5.42	61.26 ± 1.20	0.006	< 0.001	0.214
Acetic:propionic	2.53 ± 0.42	2.55 ± 0.31	2.14 ± 0.12	2.24 ± 0.21	0.743	0.073	0.817

*p* values are obtained from two-way ANOVA for the main effects of QUE (TG1), GRA (TG2), and their interaction (TG1 × TG2). Differences were considered statistically significant at *p* < 0.05. TVFA, total volatile fatty acids; A/P, acetic acid to propionic acid ratio.

### 3.6 Microbial composition of the rumen contents

#### 3.6.1 Dilution curves of the rumen microbiota

Based on the coverage index of alpha diversity, rarefaction curves were plotted to illustrate the sequencing coverage of samples in each group (Figure 1). The curves of all samples gradually increased and then leveled off, suggesting that the sequencing depth was adequate to cover most microbial species in the samples.

**Figure 1:**
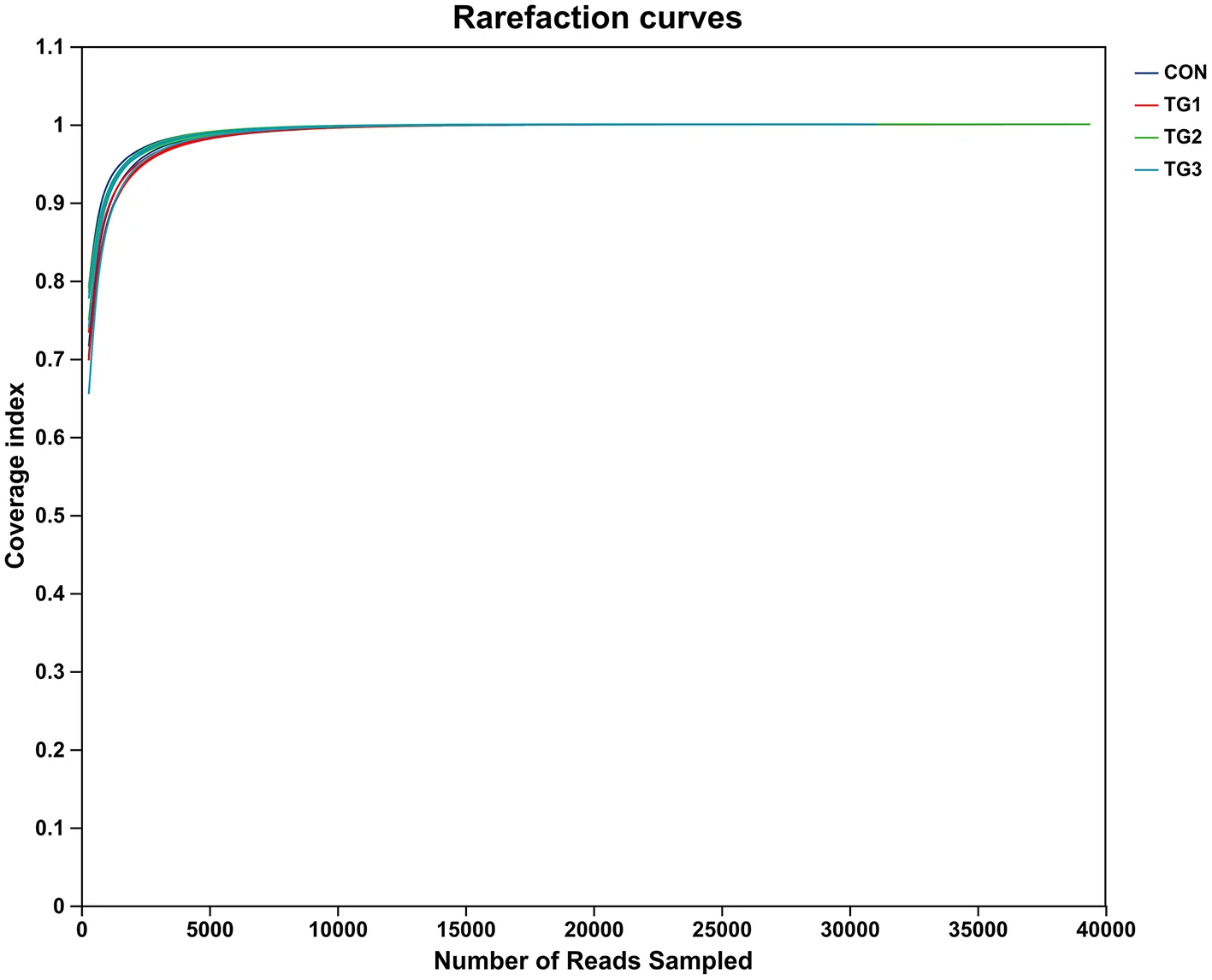
Coverage index as a function of the number of reads.

#### 3.6.2 Alpha diversity analysis

The alpha diversity analysis showed that dietary supplementation with QUE and GRA had no significant effect on microbial diversity (Figure 2; *p* > 0.05).

**Figure 2:**
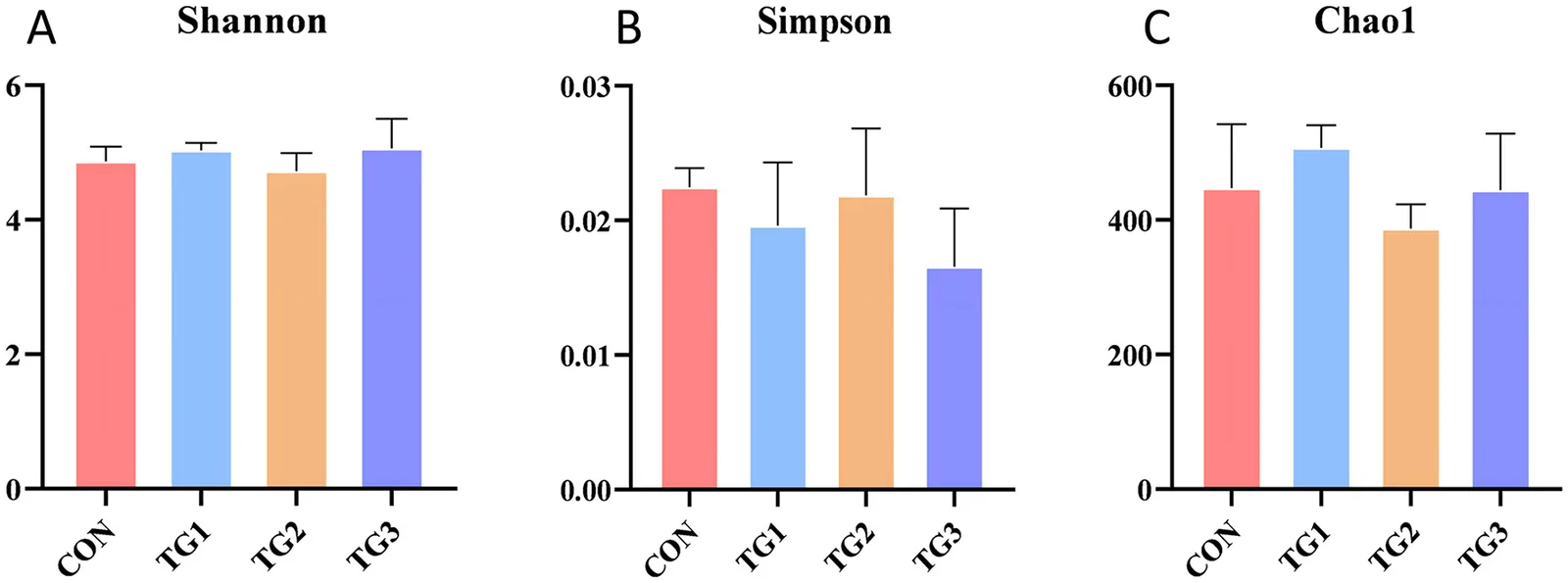
Alpha diversity analysis: **(A–C)** Shannon, Simpson, and Chao1 indices were used to assess alpha diversity.

#### 3.6.3 Beta diversity analysis of the rumen microbiota

PCoA was conducted based on the Bray-Curtis distance (Figure 3). PC1 and PC2 explained 17.06% and 14.42% of the total variation among samples, respectively (*R* = 0.3920, *P* = 0.013).

**Figure 3:**
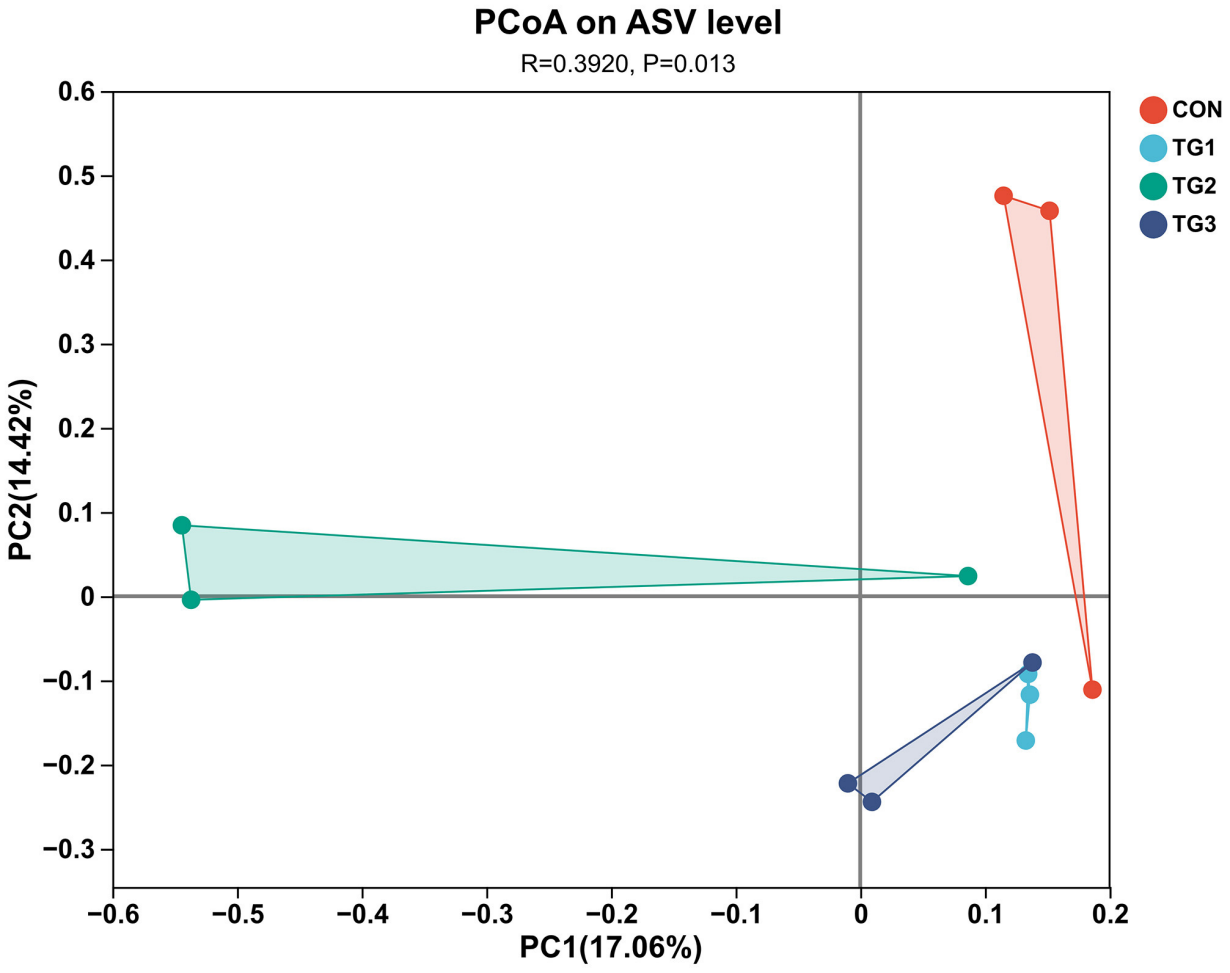
Principal coordinate analysis (PCoA) plot based on the Bray–Curtis distance matrix.

#### 3.6.4 Microbiota in ruminal contents

QUE exerted significant main effects on the relative abundances of *Xylanibacter, Succiniclasticum*, and *Ruminococcus* among ruminal microorganisms (*p* < 0.05). Furthermore, significant interactive effects between QUE and GRA were observed for the relative abundances of *Succiniclasticum* and *Ruminococcus* in ruminal microorganisms (Figure 4; Table 7; *p* < 0.05).

**Figure 4:**
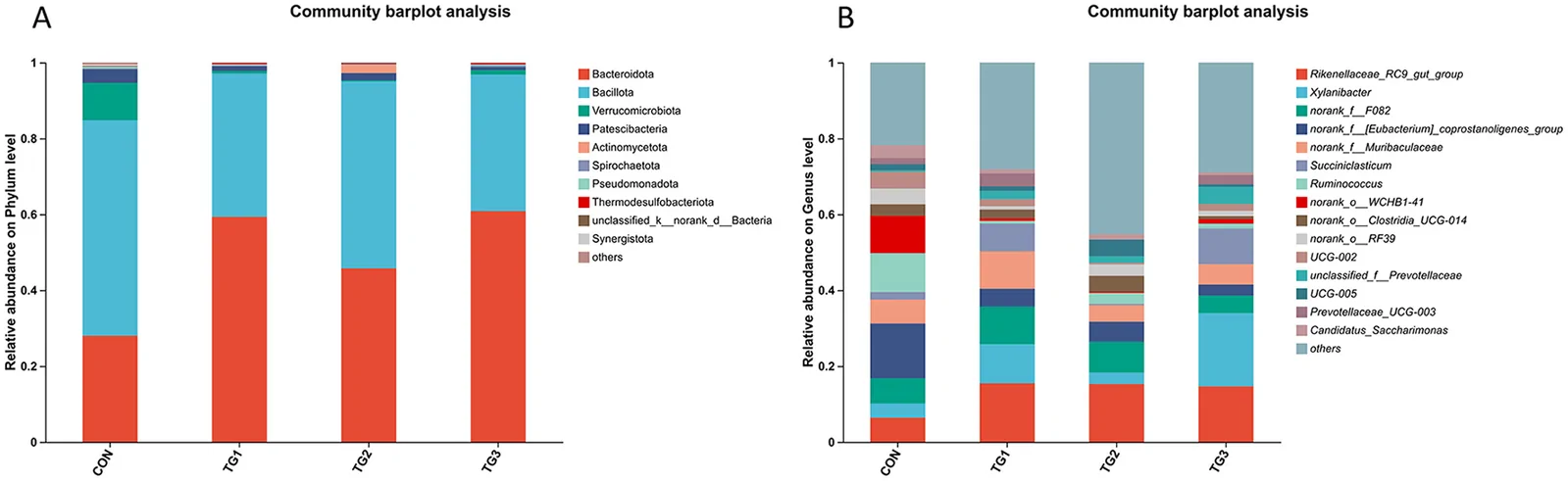
Effects of dietary supplementation with quercetin and glucoraphanin on the ruminal microflora of meat sheep (*n* = 3 per group). Relative abundances of bacterial communities at the **(A)** phylum level and **(B)** genus level.

**Table 7:** Effects of dietary quercetin and glucoraphanin supplementation on genus-level composition and relative abundance of ruminal bacterial communities in meat sheep (*n* = 3 per group).

Item	Groups				Adjusted *p* value		
	CON	TG1	TG2	TG3	TG1	TG2	TG1 × TG2
*Rikenellaceae_RC9_gut_group*	6.60 ± 5.72	15.50 ± 2.35	15.61 ± 5.03	14.63 ± 3.59	0.094	0.090	0.094
*Xylanibacter*	2.92 ± 1.66	10.41 ± 2.33	3.61 ± 2.50	19.40 ± 3.97	< 0.001	0.090	0.074
*norank_f__F082*	6.68 ± 0.46	9.90 ± 3.20	8.34 ± 5.15	4.57 ± 1.87	0.887	0.522	0.161
*[Eubacterium]_coprostanoligenes_group*	13.91 ± 11.52	4.67 ± 0.87	5.71 ± 4.93	2.84 ± 1.90	0.184	0.416	0.496
*norank_f__Muribaculaceae*	6.53 ± 8.61	9.60 ± 12.80	4.71 ± 4.59	5.40 ± 1.37	0.762	0.688	0.805
*Succiniclasticum*	2.11 ± 0.28	5.19 ± 0.81	0.30 ± 0.32	8.73 ± 1.21	< 0.001	0.202	0.001
*Ruminococcus*	10.34 ± 1.81	0.72 ± 0.24	3.21 ± 3.80	1.30 ± 0.98	0.008	0.090	0.045
*norank_o__WCHB1-41*	10.17 ± 15.27	0.64 ± 0.23	0.33 ± 0.58	1.21 ± 0.51	0.427	0.522	0.364
*norank_o__Clostridia_UCG-014*	2.90 ± 2.62	2.34 ± 0.36	4.30 ± 2.62	0.77 ± 0.34	0.146	0.941	0.312
*norank_o__RF39*	3.95 ± 3.31	0.90 ± 0.72	2.99 ± 1.79	1.35 ± 0.80	0.122	0.907	0.599

Adjusted *p* values were obtained after Benjamini–Hochberg FDR correction based on the results of two-way ANOVA for the main effects of QUE (TG1), GRA (TG2), and their interaction (TG1 × TG2). Statistical significance was set at *p* < 0.05.

## 4 Discussion

Growth performance in meat sheep is a key indicator for evaluating dietary nutrient levels, feed utilization efficiency, and economic returns in farming operations, with ADG, ADFI, and F/G as the primary criteria. The present study found that dietary supplementation with QUE exerted significant main effects on increasing ADG and decreasing F/G in meat sheep. Previous studies have reported that QUE supplementation alleviated hepatic inflammation and improved ADG in black carp fed a high-fat diet (20). Additionally, QUE supplementation has been shown to significantly increase ADG and reduce F/G in broilers by improving the intestinal morphology and the composition of the gut microbiota, thereby enhancing nutrient absorption (21), results consistent with the present study. The improvement in growth performance of meat sheep may be attributed to QUE's ability to upregulate the transcription and expression of immune-related genes, thereby modulating immune function, enhancing the resistance to pathogen invasion, and maintaining normal intestinal nutrient absorption capacity, ultimately leading to enhanced growth performance (22). In addition, dietary GRA supplementation had significant main effects on decreasing ADFI and F/G in meat sheep, which might be attributable to its metabolite SFN activating the Nrf2-mediated antioxidant defense pathway, potentially alleviating oxidative stress and improving metabolic efficiency (23). Interestingly, combined QUE and GRA treatment exerted significant interactive effects on ADFI, implying potential interplay between the two additives in feed-intake regulation, though the underlying mechanism warrants further investigation.

Meat quality directly affects the flavor, consumer acceptance, commercial value, and nutritional quality of meat and serves as an essential indicator of livestock meat production performance. It is commonly evaluated via physicochemical indices, including pH value, drip loss, and cooking loss (24). Muscle pH affects meat tenderness, flavor, and water-holding capacity and thus acts as a key determinant of meat quality. Blood circulation and neurohumoral regulation cease after slaughter and exsanguination. Muscle metabolism shifts from aerobic respiration to anaerobic glycolysis, leading to accumulation of lactic acid and a sharp decline in muscle pH, which adversely affects meat color and sensory quality (25). The results of the present study indicated that dietary QUE or GRA supplementation exerted significant main effects on muscle pH at 24 h post-mortem in meat sheep. The results indicated that dietary QUE or GRA supplementation effectively modulated post-slaughter muscle acidification and slowed the rate of pH decline in meat sheep. Similar studies have demonstrated that QUE restrained the rapid decline of breast muscle pH and improved meat quality by enhancing the water retention capacity in broilers (26). The stabilization of muscle pH and effective regulation of lipid metabolism may be attributed to QUE suppressing oxidative stress and preserving muscle cell integrity; this slows the post-mortem glycolytic rate and prevents excessive pH decline (27). SFN, the metabolite of GRA, may curtail lactate accumulation and delay the decline in muscle pH by improving mitochondrial biogenesis and function (28, 29). Furthermore, dietary QUE supplementation exerted a significant main effect on reducing intramuscular fat content in meat sheep. Related studies have demonstrated that dietary QUE supplementation alleviates the insulin resistance and inflammation induced by a high-fat diet, modulates the gut microbiota, and thereby reduces systemic fat accumulation (30). Additional studies have shown that quercetin reduces fat content and improves lipid metabolism by modulating the gut microbiota and the AMPK/PPAR signaling pathway in broilers (31). Mechanistically, QUE upregulates the PI3K/PKB signaling pathway to promote AMPKα1 expression, thereby activating the AMPK pathway. This results in down-regulation of ACC, a key rate-limiting enzyme for fatty acid synthesis, while concurrently upregulating CPT1 to facilitate fatty acid entry into mitochondria for β-oxidation and achieving bidirectional regulation of lipid metabolism (32). It is noteworthy that activation of the AMPK signaling pathway not only decreases lipid accumulation but also retards post-mortem glycolysis, which may at least partially explain the higher muscle pH in sheep supplemented with QUE.

Serum biochemical parameters in animals reflect the nutritional and metabolic status of the body, and they are important indicators of physiological function (33). ALT is a biomarker of hepatocyte injury (34). LDH is a key enzyme in glycolysis. When cell membranes are damaged or oxidative stress is exacerbated, intracellular LDH is released into the bloodstream, resulting in elevated serum LDH levels. Therefore, LDH is considered a non-specific marker of tissue injury (35). In the present study, significant interactive effects between QUE and GRA were observed on serum ALT activity in meat sheep, which may suggest possible alleviation of hepatocyte injury and improvement in liver function. Previous reports have shown that supplementation with GRA or QUE individually lowers serum ALT in mice with high-fat-diet-induced hepatic injury (36, 37). By contrast, our study conducted in healthy meat sheep implies potential interplay between QUE and GRA under non-pathological physiological conditions. This effect may be attributed to GRA's ability to upregulate antioxidant molecules, thereby eliminating reactive oxygen species in hepatocytes and mitochondria, restoring the structure and respiratory capacity of impaired mitochondria, and safeguarding hepatocyte membranes against oxidative stress-mediated injury, ultimately leading to enhanced hepatic function (38). Additionally, dietary supplementation with QUE significantly reduced LDH levels, suggesting that dietary QUE can enhance the antioxidant capacity of the animals, alleviate cellular damage, and thereby maintain systemic homeostasis in the body. Other studies have confirmed that quercetin reverses the elevation of serum LDH and alleviates cell injury in adriamycin-induced rat models (39). The protective effects observed in meat sheep may be attributed to QUE's ability to inhibit the activation of the NF-κB signaling pathway, thereby reducing the release of inflammatory cytokines and stabilizing the cell membrane structure, thereby attenuating tissue damage and maintaining physiological equilibrium (40).

Under normal physiological conditions, the generation and elimination of reactive oxygen species maintain a dynamic equilibrium. Excessive accumulation of free radicals exceeding the body's scavenging capacity induces oxidative stress and subsequent cellular damage (41). Antioxidant enzymes such as SOD and GSH-Px serve as core defenses against oxidative damage, and their activities are vital indicators reflecting antioxidant capacity (42). The results of the present study suggest that dietary QUE supplementation exerted a main effect on increasing serum SOD and GSH-Px levels in meat sheep. Furthermore, QUE and GRA exerted a significant interactive effect on serum GSH-Px activity. Previous studies have shown that dietary supplementation with QUE elevates hepatic SOD and GSH-Px activities, which in turn mitigates liver injury in fish (43). Additional studies have demonstrated that GRA alleviates high-fat diet-triggered liver damage by regulating oxidative status in mice (44). The enhanced antioxidant status may partially explain the reduced ALT and LDH levels discussed above, as mitigation of oxidative stress protects hepatocytes and other tissues from injury. From a mechanistic perspective, the unique chemical structure of QUE may enable it to function as an effective chain-breaking antioxidant. It may directly capture and neutralize reactive oxygen species and free radicals, inhibit the propagation of oxidative chain reactions, and reduce systemic oxidative damage (45). Meanwhile, the bioactive metabolite of GRA, SFN, may upregulate endogenous antioxidant enzyme expression through activation of the Nrf2 signaling pathway, thereby reinforcing the antioxidant defense system and alleviating oxidative stress (46). A significant interactive effect of QUE and GRA on serum GSH-Px activity was detected, suggestive of potential complementary modes of action of the two compounds. However, the exact molecular interactions behind this phenomenon require further exploration.

The rumen is a unique digestive organ, and a stable ruminal microenvironment is essential for animal health. Indicators including pH, ammonia nitrogen (NH_3_-N), and total volatile fatty acids (TVFA) can reflect ruminal fermentation characteristics and can be used to evaluate rumen homeostasis (47). In the present study, dietary QUE supplementation had a significant main effect on decreasing ruminal NH_3_-N concentration in meat sheep. This observation implies that QUE may facilitate more efficient conversion of ammonia nitrogen into microbial protein by rumen microorganisms, reduce excessive ammonia accumulation and absorption, and thus improve nitrogen utilization efficiency in the rumen of meat sheep. *In vitro* rumen fermentation studies have demonstrated that QUE decreases the NH_3_-N content by restraining excessive protein degradation, inhibiting deamination of branched-chain amino acids, and reducing the abundance of protozoa (48). QUE may modulate the structure and function of the ruminal microbiota, improve the fermentation environment, and promote the production of total volatile fatty acids (49). The concentration and composition of volatile fatty acids (VFA) are pivotal indicators of ruminal fermentation, dietary nutritional value, and animal productivity (50). VFAs supply more than 70% of the energy. for ruminants and are produced from dietary carbohydrates (51). Acetic, propionic, and butyric acids dominate ruminal VFA, with valeric, isobutyric, and isovaleric acids detected in minor proportions (52). Under the present experimental conditions, dietary QUE supplementation had significant main effects for higher ruminal acetate and TVFA concentrations in meat sheep. Studies have shown that when QUE was used as a fermentation substrate in the *in vitro* gas production technique, it increased acetate production (53), which is consistent with the results of the present study. It is therefore hypothesized that the stimulatory effect of QUE on ruminal fermentation may partly stem from its prebiotic-like characteristics, through which it modulates the structure and function of the ruminal microbiota, enriches beneficial microbial populations, and optimizes the fermentation milieu, ultimately affecting TVFA production (54). Furthermore, dietary GRA supplementation had significant main effects on elevating ruminal acetate, propionate and TVFA concentrations in meat sheep. This indicates that GRA can improve ruminal fermentation function in meat sheep, facilitate carbohydrate degradation, enhance ruminal acid-producing capacity, and help supply more available metabolic energy for the host. To date, few studies have investigated the effects of GRA on volatile fatty acids in ovine rumen fluid. Accordingly, it is speculated that GRA may enrich ruminal short-chain-fatty-acid-producing bacteria and reshape the ruminal microbial community structure. This can further strengthen carbohydrate metabolism and promote short-chain fatty acid (SCFA) synthesis, thereby maintaining ruminal microbiota homeostasis and improving overall fermentation activity (55). Taken together, these speculations suggest that dietary supplementation with either QUE or GRA can ameliorate ruminal fermentation and boost the ruminal degradation and utilization of carbohydrates. In the present study, no significant interactive effects between QUE and GRA were observed for ruminal fermentation-related indicators, suggesting that the beneficial fermentation effects of combined supplementation were not superior to individual supplementation.

Rumen microbial community homeostasis is an important foundation for ensuring normal rumen fermentation in ruminants. The results of alpha-diversity analysis in this study showed that dietary supplementation with QUE and GRA did not significantly alter the Chao1, Shannon and Simpson indices, indicating that QUE and GRA supplementation did not change the overall species richness and diversity of the ruminal bacterial community. Beta-diversity analysis based on the Bray-Curtis distance revealed a moderate separation trend among groups (*R* = 0.3920, *P* = 0.013). This suggests that although the overall number of species and richness of the community remained unchanged, the redistribution of relative abundances of pre-existing microbial taxa led to a moderate shift in the structure of the ruminal bacterial community. Therefore, the changes in community structure observed in the present study did not originate from gain or loss of species, but reflected an internal reorganization of existing microbial taxa. Ruminal microbiota interact closely with the host and play a vital role in nutrient absorption and energy utilization. At the phylum level, Bacillota and Bacteroidota were the predominant phyla in the rumen of Hu sheep, consistent with our findings (56). Bacillota accounted for a large proportion of the ruminal bacteria. Bacillota are responsible for degrading complex plant fibers into VFAs, and thus, they can improve the ruminal fermentation efficiency (57). Bacteroidete have crucial functions in the degradation of non-fibrous carbohydrates and proteins, providing energy for ruminants and enhancing dietary nitrogen utilization (58). The above findings illustrate how these two dominant phyla regulate metabolism to adapt to ruminants' digestive characteristics. At the genus level, the results revealed that QUE exhibited a significant main effect on increasing the relative abundances of *Xylanibacter* and *Succiniclasticum* in ruminal microbiota. *Xylanibacter* is a core microbial taxon in the rumen, primarily responsible for degrading plant fiber and providing energy to the host (59). *Succiniclasticum* facilitates fiber utilization by converting succinic acid derived from ruminal fiber degradation into propionate, which serves as the primary precursor for gluconeogenesis in ruminants (60). Significant interactive effects between QUE and GRA were observed for the relative abundance of *Succiniclasticum* within ruminal microbiota, while the relative abundance of *Xylanibacter* showed an increasing trend. We speculate that *Xylanibacter* and *Succiniclasticum* may exert interactive effect: the former degrades hemicellulose and xylan to produce succinic acid, which is subsequently converted to propionate by the latter. Synchronous enrichment of both genera may strengthen the metabolic coupling between fiber degradation and propionate production, enhancing fibrous polysaccharide utilization and propionate yield, thereby improving energy-utilization efficiency and potentially reducing ammonia nitrogen and methane emissions (61, 62). This speculation is based on the association between the known functions of these genera and changes in their relative abundances, and further validation via metabolic flux analysis or *in vitro* co-culture assays is required. *Ruminococcus* is a key fibrolytic bacterium in the rumen that breaks down the structural polysaccharides of plant cell walls, yielding VFA (63). In the present study, dietary supplementation with QUE and GRA exerted a significant interactive effect on the relative abundance of *Ruminococcus*, while no adverse effects on ruminal fermentation were observed. The enrichment of *Succiniclasticum* may compensate for the loss of fibrolytic capacity resulting from the decreased abundance of *Ruminococcus* and maintain overall fermentation efficiency via alternative metabolic pathways. This possibility requires further verification through experiments such as *in vitro* rumen fermentation assays and functional gene quantification. Collectively, QUE and GRA did not markedly alter the species composition of ruminal microbiota. They mainly modified the composition pattern of the ruminal bacterial community by regulating the relative abundances of several core functional genera, which may help sustain the stability of ruminal fermentation.

## 5 Conclusions

Dietary supplementation with QUE and GRA exerted significant interactive effects on ADFI, serum ALT activity, serum GSH-Px activity, and the relative abundances of ruminal *Succiniclasticum* and *Ruminococcus*. These findings provide new clues for exploring the potential interaction between QUE and GRA in modulating microbial community composition and systemic antioxidant status in meat sheep. The results of this study provide a scientific basis for promoting sustainable antibiotic-free ruminant production and lay a theoretical foundation for the development of safe, efficient, and environmentally friendly natural feed additives. However, this study has certain limitations: only three animals per group were used for slaughter sampling, serum analysis, and microbial sequencing, resulting in a relatively small sample size; only one combined supplementation dose was used, and the feeding trial was conducted over a short period. Future research should further verify the synergistic mechanisms and application efficacy of QUE and GRA by expanding the sample size, conducting gradient dosage trials, and prolonging the feeding duration.

## Data Availability

The 16S rRNA gene sequencing data have been deposited in the NCBI Sequence Read Archive (SRA) under BioProject accession number PRJNA1517978. The other datasets generated and analyzed during this study are available from the corresponding author upon reasonable request.
